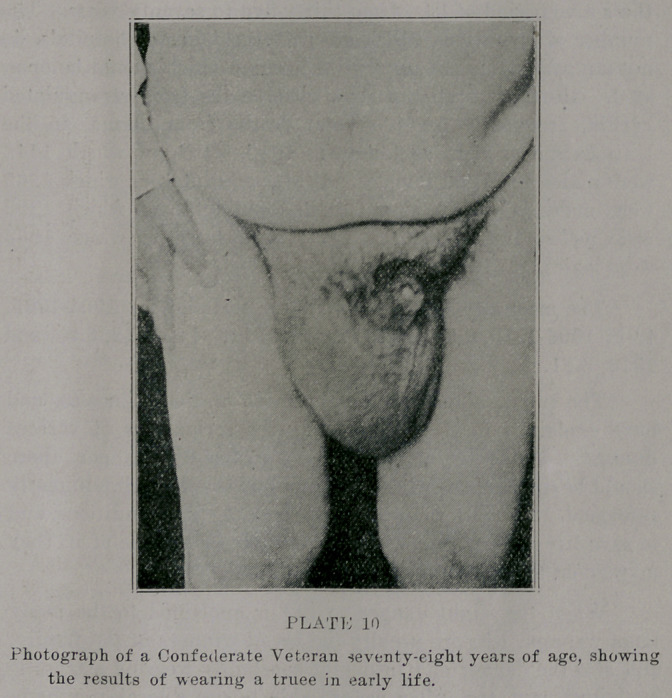# Strangulated Hernia with Operation Described and Illustrated

**Published:** 1916-03

**Authors:** L. C. Fischer

**Affiliations:** Atlanta, Ga.


### Journal-Record of Medicine

Successor to Atlanta Medical and Surgical Journal, Established 1855
and Southern Medical Record, Established 1870

**OWNED BY THE ATLANTA MEDICAL JOURNAL COMPANY**

PUBLISHED MONTHLY

*Official Organ Fulton County Medical Society, State Examing Board, Atlanta, Birmingham and Atlantic Railroad Surgeon's Association, Chattahoochee Valley Medical and Surgical Association, Etc.*

RICHARD R. DALY, M. D., Editor
BERNARD WOLFF, M. D., Supervising Editor
A. W. STIRLING, M. D., C. M., D. P. H.; J. S. HURT, B. Ph., M.D.
GEO. M. NILES, M. D., W. J. LOVE, M. D., (Ala.); Associate Editors
E. W. ALLEN, Business Manager

**COLLABORATORS**

W. F. WESTMORELAND, M. D., General Surgery
F. W. McRAE, M. D., Abdominal Surgery
ALLEN H. BUNCE, Medical Societies
E. B. BLOCK, M. D., Diseases of the Nervous System
MICHAEL HOKE, M. D., Orthopedic Surgery
CYRUS W. STRICKLER, M. D., Legal Medicine and Medical Legislation
E. C. DAVIS, A. B., M. D., Obstetrics
E. G. JONES, A. B., M. D., Gynecology
ALLEN H. BUNCE, M. D., Pathology and Bacteriology
R. T. DORSEY, JR., B. S., M. D., Medicine
L. M. GAINES, A. B., M. D., Internal Medicine
GEO. C. MIZELL, M. D., Diseases of the Stomach and Intestines
L. B. CLARKE, M. D., Pediatrics
EDGAR PAULLIN, M. D., Opsonic Medicine
THEODORE TOEPEL, M. D., Mechano Therapy
R. R. DALY, M. D., Diseases of the Eye, Ear, Nose and Throat
BERNARD WOLFF, M. D., Diseases of the Skin
E. G. BALLENGER, M. D., Diseases of the Genito-Urinary Organs

VOL. LXII    ATLANTA, GA., MARCH, 1916.    NO. 12

### STRANGULATED HERNIA WITH OPERATION DESCRIBED AND ILLUSTRATED

BY L. C. FISCHER, M. D., Atlanta, Ga.

Of the many surgical conditions that should be kept constantly in the minds of Physicians, hernia is one of the most important. While wonderful improvements have been made surgically, in the upper abdomen in recent years, hernia has to a certain extent been neglected. I know of no pathological condition that is so sure of bad results without attention, and none that is so successful with the proper care.

When a hernia becomes strangulated and is not reducible by mild taxis, I say mild advisedly, then surgical interference is the only relief. Taxis, however, should not be practised if the strangulation has existed for twenty-four hours or more; the possibility of gangrene of the herniated intestines, and almost certain trauma of the soft parts, are indications for imediate surgical interference. Without operation, death is inevitable. In these conditions the symptoms rapidly grow alarming.

PLATE 1

Showing the relations of the External Oblique, Poupart's ligament and spermatic cord with superior and inferior columns of External ring, and the inter columna fascia.

In January, 1914, I reported in the Journal-Record of Medicine one hundred and forty-eight herniotomies with description of an operation without a death, and up to that time had only had two recurrences. Thirty of the cases reported were strangulated at time of operation.

Have recently investigated and have been able to hear from eighty-four of these cases. Up to this time have had no recurrences except those reported. The shortest time since operation in this series has been more than two years. In that paper, I described an operation which is very similar to the Andrews radical cure for hernia, with some slight modifications that are valuable.

PLATE 2

Showing the most effective way of protecting the wound with towels, fastened on with clips so that there may be less possibility of contaminating the tissues not directly involved in the operation. Also the incision to be made in the External Oblique muscle well above Poupart's ligament

Strangulation may occur at any age or stage of the development of hernia. The youngest strangulated case operated upon, was a babe of four months. The oldest, a man eighty-seven, with double strangulation, and this congenital. He remembered that he had worn a truss since he was a boy of eight. The amount of gut involved in the strangulation will vary from a very small knuckle to any quantity of intestines that are allowed to be, or is caught in the stricture. The seriousness of the condition is not influenced to any extent by the size of the tumor. Some of the most serious and dangerous conditions are those where the mass is very small and almost imperceptible, except upon very close examination, and, therefore, often being overlooked until the patient's conditions is too extreme for operative relief.

PLATE 3

Showing the disposition of the sac after it is ligated and amputated, fastened well up under the Internal Oblique and transversallis muscles, presenting entirely new peritoneum to the original site of the hernia.

Since my last report, I have seen two cases too far advanced from strangulation to admit of operation. One a female with femoral hernia that had existed for twenty-four years, and had been strangulated for six days at the time I saw her. She having general peritonitis with an imperceptible pulse. She lived in the country and had not called her physician until the day I met him there. The second case, a man fifty-six years old with congenital hernia. He stated that for years he had tried to get an operation done, but was told that on account of the enormous size of the rupture, and a bad heart, an operation was impossible. This strangulation occurred while he was wearing a truss which had always been comfortable up to about six years ago, when it had been impossible to get one that would hold the mass up. Taxis was practised by his doctor who was an osteopath. There was so

PLATE 4.

Showing the normal relations of the Internal Oblique and Transversallis muscles, the conjoined tendon, the spermatic cord, and showing the normal arch around the internal ring of these structures, also Ilio-Inguinal Nerve.

much injury done at the time that there was a laceration of the soft parts including the external oblique. The edema and ecchymosis were so pronounced at the time, one would be excusable for thinking the mass an abscess. He had vomited continuously for five days, with complete obstruction. With a local anesthetic, an incision was made down to the hernial sac. Upon opening this its entire contents were necrosed, which consisted of the cecum, appendix, portion of ascending colon, and what appeared at the time to be the greater portion of the small intestines.

While I do not feel that under many circumstances, the surgeon has the right to refuse to operate, in these two cases, I felt that any radical procedure would be foolish. It is our most sacred duty, and one I have tried to live up to, if in our judgment, the patients have the slightest chance for recovery, we should give them the benefit of the doubt and operate, and not shirk such a condition in order that our operative mortality may be low.

The treatment of strangulated hernia should be promptly and carefully carried out. Taxis in the hands of most men is dangerous; still this is the only course left open to us without an operation. It should be very gently done, with patient in reclining position, hips slightly elevated, thighs flexed, and an anesthetic administered if necessary. The first effort is to empty the gut, this should gradually reduce the mass into the abdomen. This effort should not be persisted in until injury is done, but operation should be resorted to at once. The point of constriction is usually in the external ring, or the neck of the sac. At operation the gut should by no means be reduced, after being strangulated for a few hours, without careful inspection; as in twenty-four hours if there is not an actual gangrene there may be such pressure on the bowels as to produce a paresis that the intestines will not overcome even after the constriction is removed, and in this case there will still be symptoms of intestinal obstruction, and the patient die just as surely as if there was actual gangrene, or the strangulation was not removed.

I do not claim any originality for this operation, though I had worked it out independently of the operations of others, and it was only after I had been doing it for several years that I found out that Andrews was doing and had reported the same operation, except a slight modification in the final closure.

The preparation of the patient does not vary from that for any other abdominal operation: infection, however, in this wound means much more to the patient, and in the larger majority of cases, results in a recurrence; if not immediate, in years to come·

PLATE 5

Showing the closure of the Internal Oblique, Transversallis and conjoined tendon below the cord to the shelving ledge of Poupart's ligament, bringing the cord out at the extreme upper angle. where Internal Oblique and Transversallis take their origin from Poupart's ligament.

The location of the incision (plate 1), the dissection of the sac, the isolation of the cord, etc., does not differ from that of the same procedure in the many other operations for radical cure. In this as in all abominal operations, it is wise to wall off with towels or gauze (plate 2) after making the incision, the skin and all soft parts down to the muscles, thereby preventing the possibility of contamination of the deeper structures by germs, always present in the skin·

After ligating and cutting off the sac, the stump is fixed well up under the conjoined tendon which brings entirely new peritoneum over the inguinal canal (plate 3). Some of the best authorities have stated, that in femoral hernias especially, ligation of the sac is all that is necessary to make a complete cure. I do not agree with these radical ideas, at the same time, the care of the sac or stump means much. Should the sac be left undis-

PLATE 6

Showing the upper portion of the External Oblique closed down to Poupart's ligament, under the cord with the partial formation of an external ring, directly over the Internal ring at the extreme upper angle of the wound.

turbed, even though the muscles are closed properly, there will almost certainly be a recurrence, due in most cases to the same forces that produced the original condition. The pressure of the abdominal contents will surely follow the old sac, acting as a wedge, which will gradually enlarge the new formed ring. After disposing of the stump of the sac, as above described, make a clean distinct dissection of the cord and muscles, (taking great care to isolate the ilio-inguinal nerve plate 4). Failure to do this properly is one of the greatest causes to allow or produce recurrence. All of the tissues in the inguinal region must be so dissected, that each individual one is recognized. After this, retract the divided edges of the aponuerosis of the external oblique, together with the ilio-inguinal nerve, so that the conjoined tendon, and the muscles that compose it, are well exposed, both in the insection of the conjoined tendon into the crest and ilio-pectineal line of the os pubis, the arching fibers of the two muscles, also the origin of the muscles from Poupart's Ligament (plate 4). With the cord retracted, close the conjoined tendon and the arching fibers of the internal oblique and transversallis (plate 5) under the cord to the under margin of the gutter-like process or portion of Poupart's ligament. Beginning at the insertion of these into the spine, crest and ilio-pectineal line, close them out to the original internal ring, then with the cord making its exit at this point, place two or three stitches external to the cord. This forms an entirely new ring. Failure to take these stitches above the cord or to the outer side of it, is liable to allow too large a ring to be formed. The origin of the muscles from Poupart's ligament is largely muscular tissues, with very little tendonous attachment. The new ring thus formed has entirely new boundaries; the lower and upper ones being Poupart's ligament and the muscles above. To form the external ring and close the external oblique (plate 6), begin at the same point, closing the uppercut edge of the aponuerosis of the external oblique, down to the same under margin of the gutter-like process of Poupart's ligament, out to the new formed internal ring, taking from one to three stitches external to the cord. Finally (plate 7) close the lower cut margin of the aponuerosis of the external oblique up over the two rows of sutures, thus overlapping the aponuerosis of the external oblique; with the lower fold plicated up over the aponuerosis of the external oblique about an inch above the two other lines of sutures. The same care should be taken to make allowance for the cord, or to complete the formation of the external ring. The overlapping is done in the same way as described by Halstead in his modification of Ferguson's operation. This closes entirely the external ring at its original site, which is the most dependent point of the abdomen. While the new rings are nearer the middle of Poupart's ligament, they are as far removed from the bony insertion of the muscles and ligaments as possible. Being placed as they are, there is more elasticity of the muscles at this point, making it possible to close more nearly the ring to the exact size of the cord; making it less likely to cause strangulation, at the same time closing entirely the most dependent point of the abdomen. All of the recurrences that I have ever seen have been in the original course of the cord. With the operation completed so far as the muscles are concerned, this gives entirely a new floor for the cord which consists of the external oblique, the conjoined tendon, internal oblique, and transversallis muscles; its

PLATE 8

**Showing the coverings of the cord with the deep and superficial fascias, making its new relations; resting on the External Oblique muscle and covered only by the deep superficial fascias and skin.**

coverings are, the deep and superficial fascias, together with the skin (plate 8). In my work the patients have not complained of as much pain in the cord and testicles following this procedure, as they have with the Bazani and Ferguson operations.

Have often said that I have never fitted a truss, and never expect to. Plate 10 is a photograph of a Confederate Veteran seventy-eight years of age, who occupies at present a place in the Soldiers' Home of this State. He at this age, very bitterly denounces his former medical advisers for not having insisted that he have an operation. He is perfectly hale and hearty, but with the enormous hernia, is completely incapacitated.

PLATE 9.

Final closure of the wound which is done with either number 1 Chromic gut, or Michael's clips.

In operating upon cases who have worn a truss for years, there are most always complications or adhesions from pressure, and in many cases a partial atrophy or atony of the muscles from having been so long supported by a truss

Dr. W. A. Selman in the Journal-Record of Medicine, August, 1915, says in describing an operation for strangulated hernia, "The intestine regained its color sufficiently to show vitality, so resection was not done. A very tedious dissection of the sac consumed a long time as a truss had been worn for years." The statement of Dr. Selman, "a very tedious dissection of the sac consumed a long time, as a truss had been worn for years," is a feeling that any surgeon can remember and so often impresses him at the time of operation.

It is amazing to see the great numbers of sufferers from hernia, in life insurance examinations, etc., who are wearing a truss, or in a great many cases, who do not; and who do not real-

PLATE 10

Photograph of a Confederate Veteran seventy-eight years of age, showing the results of wearing a truee in early life.

ize the seriousness of their condition. Keys, in Essays on Abdominal Surgery, says: "That one-fifth of all people have some form of abdominal hernia." He reports in 10200 male patients operated upon in Cook County Hospital, that 8 1-2 per cent were operated upon for hernia; that 82 1-2 of these were for either direct or indirect inguinal hernia. In 6654 females 2 1-2 had some form of abdominal hernia, and 10 per cent of which were inguinal.

Berger estimated that the proportion of ruptures in males is 1 in every 14.9 and in females 1 in 44.7. He gives the average as 1 in 22 in both sexes. The studies also showed, that there was a steady increase in males, in the number of hernias during the active period of life, from thirty-five to seventy years. The number of operations which are performed in our hospitals, do not any way equal the number of hernias which should be operated. Mortality statistics show that deaths from strangulated hernia, frequently occur. Total deaths from hernia, in the United States, were as follows: 1910, 2192, of which 1142 were males and 1050 females. 1911, 2369 total, of which 1265 were males, 1104 females. 1912, 2348 total, of which 1222 were males, 1126 females. 1913, 2424 total, of which 1263 were males, 1161 females.

The mortality rate per 100,000 is as follows: 1901-1905, 4.02; 1906-1910, 4.01; 1910, 4.01; 1911, 4; 1912, 3.9, and 1913, 3.81.

The public should be taught more of their illnesses, and have explained to them more fully the seriousness of various diseases. While we, as physicians, realize the danger, there should be some means whereby the public should be intelligently informed. No matter our ability to relieve these conditions, and to save life; unless the patient knows the seriousness of it, they may go on to old age, totally incapacitated.

When the slight danger to life is explained to the many truss wearers, who are usually the bread winners of the family, and also the danger and injury of constantly wearing a truss is explained, and, too, when we are able to use a local anesthetic in a great majority of these cases, thereby, relieving them of a fear of an anesthetic; many more of them will submit to an operation and be restored to normal manhood. As years pass on and the stage of decline approaches, it becomes more and more impossible to find a truss that will support the greatly increasing mass, and make it possible for one to be comfortable.

If operations for hernia were done earlier in fact, as early as the hernia is recognized, a great many physical weaklings would be normal men and able to compete with their fellow man in all vocations of life.

My plea is, that, more cases of hernia be operated upon when they first occur; or in youth, as we can assure them of greater safety in operation at this time. There are a great many more patients who have hernia that are not operated upon than the number cured by operation.

819 Hurt Building,

REFERENCES:

Fischer, Journal Record of Medicine, January, 1914.

Selman, Journal Record of Medicine, August, 1915.

Keys, Essays on Abdominal Surgery Wm. Wood & Co., 1915.

Jacobson, American Journal of Obstetrics and Gynecology, November, 1915.

Ferguson, Modern Operations for Hernia.

Taylor, Operative Surgery.

## Figures and Tables

**PLATE 1 f1:**
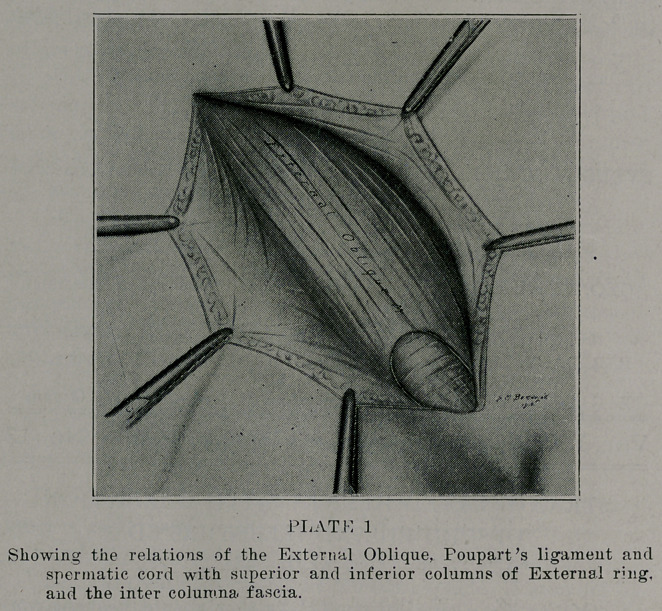


**PLATE 2 f2:**
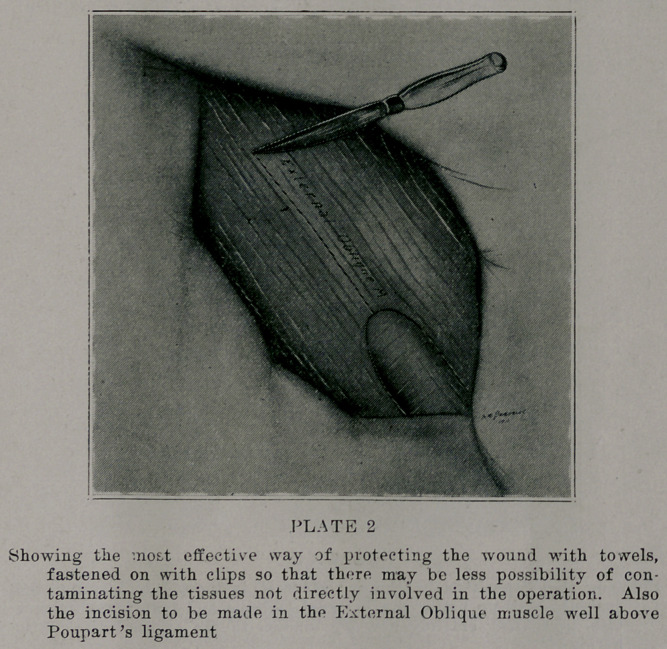


**PLATE 3 f3:**
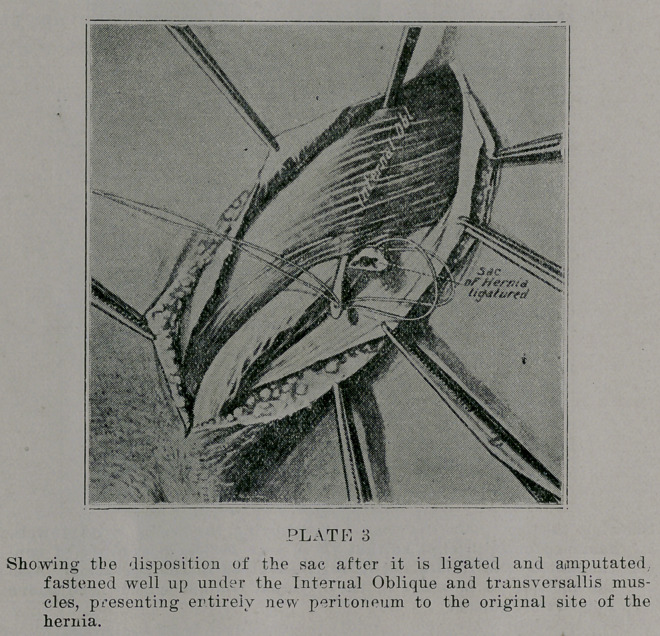


**PLATE 4 f4:**
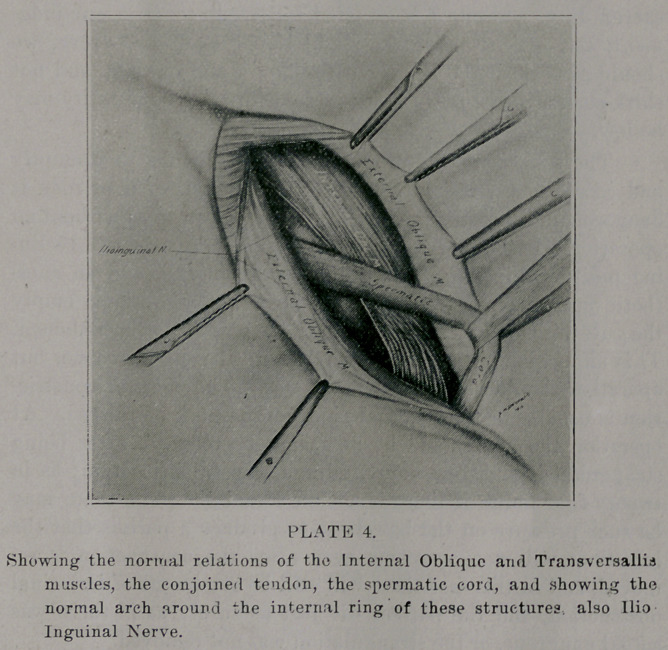


**PLATE 5 f5:**
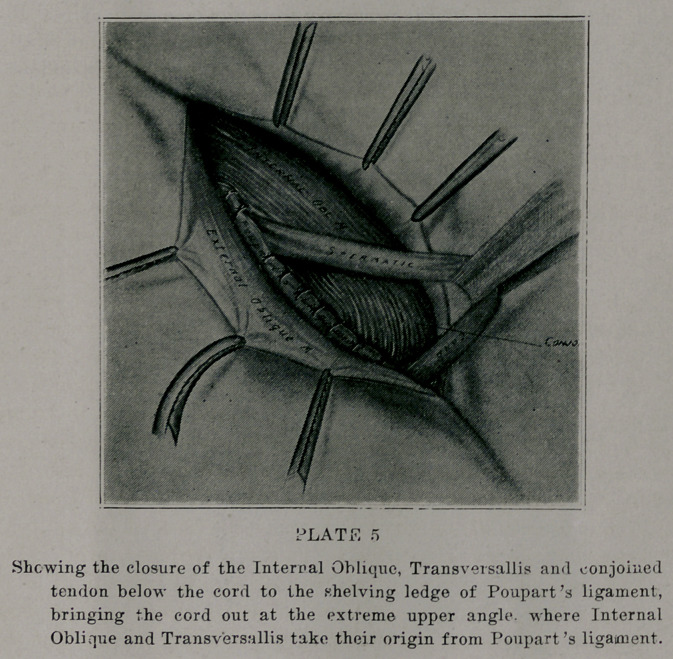


**PLATE 6 f6:**
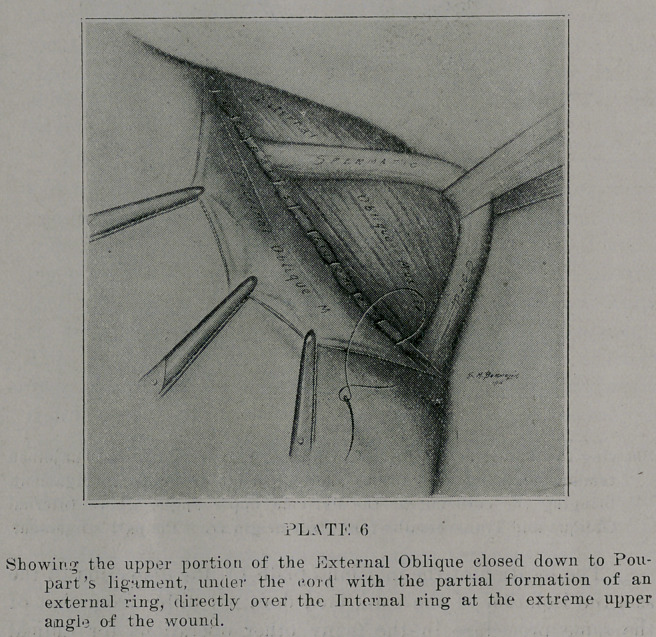


**PLATE 7 f7:**
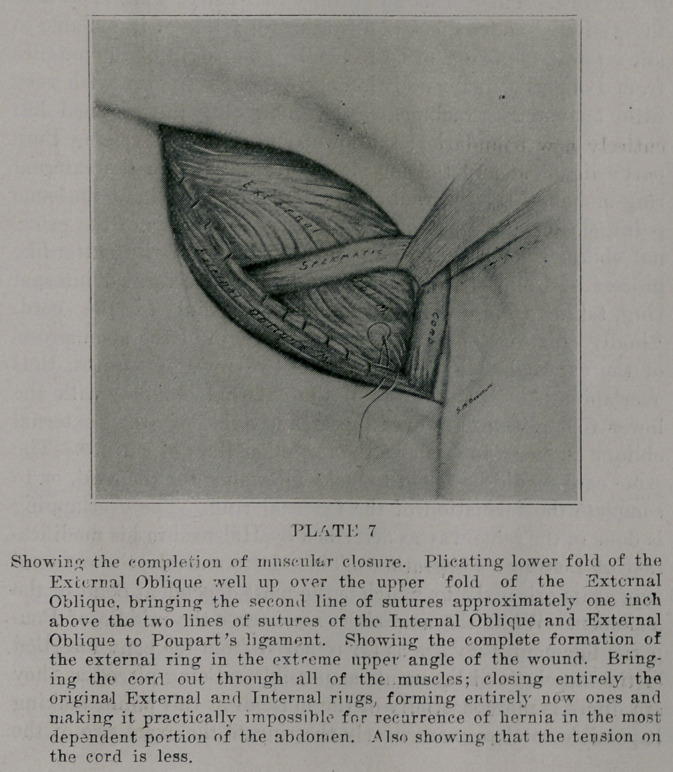


**PLATE 8 f8:**
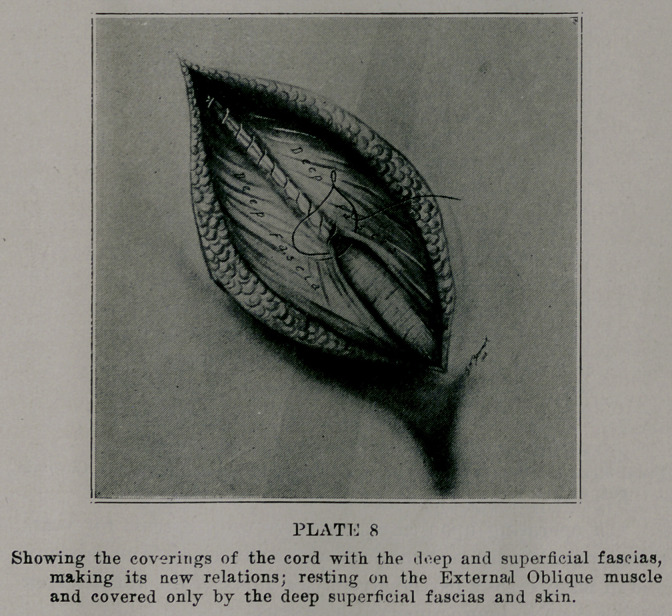


**PLATE 9 f9:**
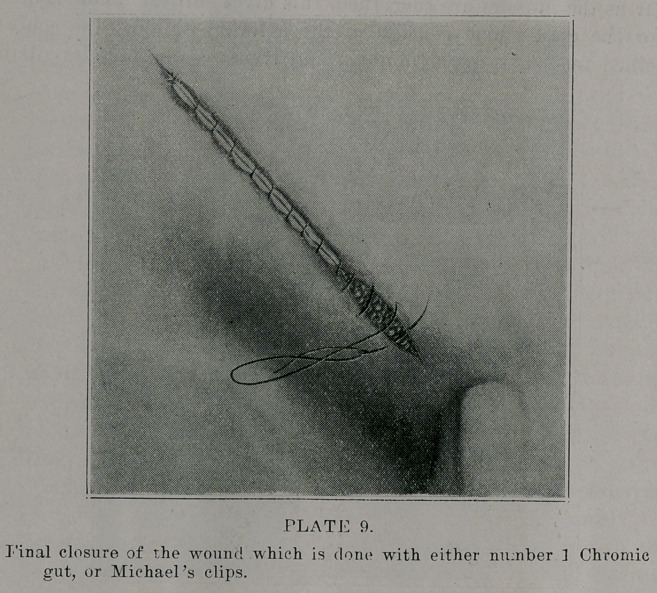


**PLATE 10 f10:**